# The effects of different sports activities on body scheme in preschoolers and primary school children: an experimental and theoretical analysis

**DOI:** 10.1186/s40101-026-00422-0

**Published:** 2026-02-11

**Authors:** Mikhail Shestakov, Elena Godina, Tamara Abramova, Alexander Korchagin

**Affiliations:** 1 Federal Science Center of Physical Education and Sport, Elizavetinsky Lane 10, Building 1, Moscow, 105005 Russian Federation; 2 Anuchin Research Institute and Museum of Anthropology, Lomonosov Moscow State University, Mokhovaya St., 11, Moscow, 125009 Russian Federation; 3 Department of Computational Mathematics and Cybernetics, Lomonosov Moscow State University, GSP-1, Leninskie Gory, Moscow, 119991 Russian Federation

**Keywords:** Postural balance, Body scheme, Active inference, Children, Sport specialization

## Abstract

**Background:**

Children are now introduced to sports at an early age, often beginning to learn complex movements between the ages of 3 and 5, which coincides with the formation of a body schema. Active inference theory suggests that the brain uses internal probabilistic models to assess the state of the body, adapting to sensory errors received from various sources. In preschool and primary school children, targeted sports training is thought to improve body awareness. We propose that targeted and prolonged sports training in preschool and primary school children develops specific features of body awareness that reflect the performance requirements of the corresponding sport.

**Main:**

Our experimental data confirmed age differences in postural oscillations during natural standing in children aged 5–9 years (*n* = 692) who were engaged in various sports, with similar data obtained by other researchers on a contingent of children not involved in sports. The studied parameters of athletes after 7 years were significantly different from those of younger children: radius (*p* < 0.001) and speed (*p* < 0.0001) of COP oscillations. The developed control model based on the active inference theory allowed identifying the body scheme as an internal cause explaining the experimental data in 8-year-old athletes with at least 3 years of experience: gymnasts have an inaccurate body scheme caused by errors in predicting the sensory effects of posture, which are compensated by motor hypercorrections; the body scheme of the team players is distinguished by balanced data from sensory sources; the inert body pattern of skiers is characterized by insufficiently accurate prediction of changes in movement conditions, which leads to energy-consuming corrections.

**Conclusion:**

Our results show that the development of body schema in preschool-aged and primary school-aged children is closely related to multisensory integration and the specificity of the actions used in systematic sports. The model experiment demonstrated that practicing a certain sport forms the appropriate proportions between the modalities, determining the features of the internal body scheme of athletes of different specializations.

## Background

The body schema is an important perceptual phenomenon that enables humans to perform everyday voluntary movements [1], including sporting activities [2]. It requires a sufficiently accurate assessment of the spatial position of the body, which is based on information from visual, vestibular, and somatosensory data [3].

The control of quiet standing is often modeled as the control of an inverted pendulum. The inverted pendulum model postulates a one-to-one relationship between estimates of the kinematic state of the body in space and estimates of the position of the ankle joint [4]. However, the inverted pendulum model is an external representation of an object, whereas the control issues of postural balance change according to changes in the internal representation of the body configuration [5]. The proposed theory of active inference (AIF) [6] considers the brain’s control decisions via internal probabilistic (Bayesian) models [7]. These models allow the brain to flexibly estimate the state of the body and the consequences of its movement through iterative updating over sensory prediction errors from multiple sources [8, 9]. Body position can be informed by signals from multiple sources of sensory information, including vision and proprioception [10]. The contribution of each modality to the overall optimal integration of signals is determined by its relative reliability or precision depending on the current context [11]. The application of active inference to postural balance reveals how high-level body representations, including body schema and body image, are involved in predictive motor and postural control [12]. Research has demonstrated that body schema and subjective evaluation of one’s own body are directly linked to concurrent sensorimotor control of body sway, suggesting that these representations play active roles in maintaining postural stability [12]. Physical activity and purposeful sports training can be considered contexts that influence the development of the ability to control one’s body while improving the perception of one’s own body [13]. Currently, children are being introduced to various sports at the age of 3–5 years. Between the ages of 3 and 5, the body schema—an internal representation of one’s own body’s position and movements in space—begins to actively develop. This is the foundation for mastering complex movements [14, 15]. This stage is characterized by the development of coordination, balance, and spatial awareness, which are closely linked to proprioception, vision, and sensorimotor integration. It is through the development of the body schema using basic sports movements that the child acquires the ability to control and plan complex movements, confirming the importance of an early start to sports. The child’s mastery of complex specialized sports movements occurs simultaneously with the formation of the body schema. Both the type of sport and the level of activity can affect balance control [16]. In typically developing children, postural sway decreases with age under different sensory conditions [17].

We hypothesize that targeted and prolonged sports activities in preschoolers and primary school children develop specific features of their own body awareness, reflecting the performance requirements of the corresponding sport.

## Materials and methods

The survey participants included 692 children: 5 years old (*n* = 98), 6 years old (*n* = 148), 7 years old (*n* = 154), 8 years old (*n* = 169), and 9 years old (*n* = 123), who were involved in gymnastics (G, *n* = 209), alpine skiing (AS, *n* = 238), and team games—football and ice hockey (TG, *n* = 245). The peculiarities of the formation of training groups by coaches determined that only boys were present in the experimental groups. The characteristics of the children participating in the experiment are presented in Table 1. The beginning of systematic training in our study in G was noted at the age of 4.88 ± 1.45 years, AS—4.75 ± 0.88 years, in TG—4.85 ± 1.21 years. The training load of children depended on the year of training. In the first year of training at the basic preparation stage, the weekly load was in groups: G—3 times for 1 h, AS—1 time for 1.5 h, TG—2 times for 1 h; 2nd year—G—4 times for 1.5 h, AS—2 times for 2 h, TG—3 times for 1.5 h; 1 st year of the sports specialization stage—G—5 times for 2 h, AS—3 times for 2.5 h, TG—4 times for 2 h; 2nd year—G—6 times for 2.5 h, AS—4 times for 3 h, TG—5 times for 2.5 h; 3rd year—G—7 times for 3 h, AS—6 times for 3.5 h, TG—6 times for 3 h.

**Table 1:** Characteristics of participants by age and sport

Age, years	Gymnastics (n)	Alpine skiing (n)	Team games (n)	Average height* (cm)	*p*-value^	Average weight* (kg)	*p*-value^	BMI* (kg/m*m)	*p*-value^
5	34	31	33	115,4 ± 4,4	0.453	21,0 ± 2,8	0.287	15,7 ± 1,4	0.512
6	47	56	45	120,9 ± 5,2	0.621	23,4 ± 3,6	0.398	16,0 ± 1,5	0.674
7	39	64	51	126,1 ± 4,9	0.785	25,7 ± 4,0	0.556	16,1 ± 1,6	0.432
8	49	61	59	130,7 ± 5,0	0.334	29,0 ± 5,2	0.219	16,9 ± 2,0	0.367
9	40	26	57	135,9 ± 5,6	0.498	31,1 ± 4,5	0.411	16,8 ± 1,6	0.523

^*^ Mean ± SD

^ *p*-value from ANOVA comparing the three sport groups within each age category

One-way ANOVA was conducted within each age group to compare the three sport groups. No statistically significant differences were found in height, weight, or BMI between sport groups within any age category (all *p*-values > 0.05).

To eliminate possible side effects, we conducted a preliminary analysis to assess differences in anthropometric characteristics (height, weight, BMI) between sports groups in each age category. One-way analysis of variance (ANOVA) was used. Since no significant differences were found (Table 1), these variables were not included as covariates in the main ANCOVA models. However, as a sensitivity analysis, we also used ANCOVA models with height, weight, and body mass index as covariates, which yielded results similar to those of the primary ANOVA models (Height: F-value = 1.23, *p*-value = 0.267, Partial η2 = 0.004; Weight: F-value = 1.23, *p*-value = 0.267, Partial η2 = 0.004; BMI: F-value = 1.23, *p*-value = 0.267, Partial η2 = 0.004).

The study was conducted using a stabilometric complex for recording data on the movement of 2-D coordinates of the center of pressure (CoP) with biofeedback “Stabilan-01” (NPO “Ritm”, Taganrog, Russia) [18] with a data sampling frequency of 50 Hz, a force measurement range from 20 to 150 kg, and a mass indication resolution of 0.1 kg. The CoP data was filtered with a Savitsky-Golay filter with window size 7. The test was performed via visual feedback, which was provided to the subject on a monitor screen located at a distance of 1.5 m and at the subject’s eye level and individually adjusted for eye height. Visual feedback was provided via a 21.5" monitor with a resolution of 1920 × 1080 and a refresh rate of 100 Hz. The CoP movement marker was displayed as a circle with a diameter of 2 degrees of visual angle without delay. This task is a model of visually controlled postural maintenance, aimed at studying the role of visual information in the context of AIF. The examination was conducted in the morning, with the condition that no vigorous physical activity should take place before the test. The subject sat quietly for 5 min before the examination, filling out personal information. Afterward, they were given instructions on how to conduct the examination, after which a trial procedure was conducted to ensure that the subject understood the instructions correctly. Data recording was performed once with a 1.5-min rest interval between the test and main attempts. According to the study conditions, the subject stands barefoot evenly on the stabiloplatform according to the European system of foot placement (with a distance of 2 cm between the heels, the toes are spread at an angle of 30°, the position of the hands in the Napoleon pose or on the hips, with hands excluding their participation in the movement. The subject must hold the marker displaying the position of his CoP on the stabiloplatform in the center of the target. The instructions stipulate that the movement should be performed exclusively in the ankle joints. The testing procedure lasted 30 s, including the data writing delay in the first 10 s [19]. The following test performance indicators were recorded: R—average spread (average radius) of the CoP deviation from the zero-point, V—average COP movement velocity, X, Y—initial CoP displacement in the sagittal and frontal directions (mathematical expectation of the CoP position coordinates), but since no significant differences were found (*p* > 0.01), they are not presented further.

Participants with neurological disorders, visual impairments, orthopedic pathologies of the lower extremities, and vestibular dysfunctions were excluded. In case of failure to fulfill the conditions of the motor task: arm movements, movements in the knee and hip joints, shuffling of the legs, lack of concentration, the data were excluded from the database. They and their parents were informed about the experimental procedures. All parents of the subjects provided informed consent before the experiment. The data are presented as the means with standard deviations.

The survey program and methodology complied with the provisions of the Helsinki Declaration and were approved by the Ethics Committee of the Federal Scientific Center VNIIFK (No. 3.23) on October 24, 2023.

Data were tested for normality using the Shapiro–Wilk test; in cases of deviation, nonparametric methods were used. Multiple comparisons were corrected using Holm’s method. Comparisons between groups were made using Cohen’s d (small d ≈ 0.2) and Student’s t-test for independent samples. Homogeneity of variances was tested using Levene’s test. In the presence of interactions, simple effects were analyzed using post-hoc tests.

Between-group comparisons were performed using two-way analysis of variance (ANOVA) with factors “sport type” and “Experience”. To control for potential confounding effects of anthropometric characteristics, analysis of covariance (ANCOVA) was conducted with height, weight, and BMI. When significant main effects or interactions were found (*p* < 0.05), post-hoc pairwise comparisons were performed using Tukey’s Honest Significant Difference (HSD) test with adjustment for multiple comparisons.

The agent model was developed in the Python 3.9 environment via the pip 3 9.0.1, openpyxl 3.1.5, SciPy 1.16.3 packages. Statistical processing of the obtained material was performed in the R-Studio 9.5 Build 191,648 environment via the ggplot2-ru 4.0.0, dplyr 1.1.4 and caret 7.0–1 packages.

### Modeling

To conduct a theoretical analysis between the obtained experimental data of performing upright standing with visual control and the reasons underlying the obtained movement result, a model based on AIF was developed [8, 9].

**Fig. 1:**
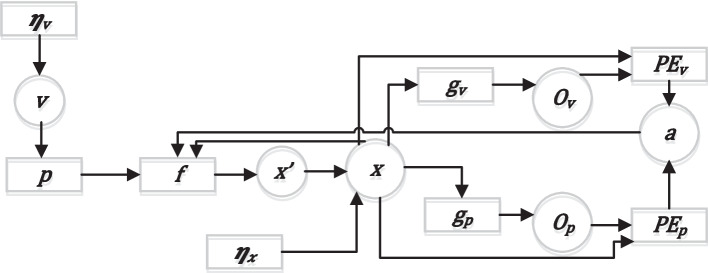
Factorial graph of an agent controlling the equilibrium of a single-joint inverted pendulum. The variables and factors are indicated by circles and squares, respectively. v—hidden cause (unobservable factors causing sensory inputs, correspond to the actual body configuration, including muscle activation); and ηηᵥ ₓ—prior beliefs (“body schema” in this context); f—dynamic function; and ₚ ggᵥ—likelihood function; x—hidden states (e.g., joint angles); and ooₚ ᵥ surveillance (e.g., visual and proprioception); ϱ attractor (Target Center); x'—derivative of the state; PEv and Pep—of the prediction error (the difference between predicted sensory inputs and actual sensory inputs); a—action (movements)

In the context of AIF, the concepts of model and agent are not synonymous. While referring to the same system, they complement each other. A model refers to an agent’s representation of the world around it. An agent is a system capable of perceiving the environment, generating predictions about it, and acting on these predictions to achieve a certain goal, using its model of the world to reduce its free energy. To perform an action, the agent selects those observations that correspond to its previous beliefs. The most basic scheme of the agent is shown in Fig. 1, which was reworked based on work [20]. This is the simplest representation with key nodes of a continuous in-time active inference agent. The scheme fully complies with the principles of active inference and can be used to model both static balance and tracking movements. The presented description generally correctly reflects the key principles of active inference [6, 11]. The model experiment was carried out for each group of sports until the corresponding values for the studied indicators were obtained, these values which did not differ significantly (*p* > 0.05) when the experimental and simulated data were compared.

In this study, the Nelder–Mead optimization algorithm was used to find the optimal model parameters by minimizing the RMSE of the radius (R) and velocity (V) parameters, implemented via the scipy.optimize.minimize function. An initial starting point was selected on a coarse grid, after which the Nelder–Mead method was applied to locate the minimum. Optimization validity was verified by a two-sample test confirming no statistically significant differences between simulated and experimental values (*p* > 0.05).

The model is deterministic given a fixed seed and parameters, ensuring reproducibility of simulations; however, various datasets were generated for testing. Sensitivity analysis involved sequentially varying each parameter by ± 5%, assessing changes in RMSE and free energy (EFE), which allowed ranking parameters by local effect.

Convergence was defined as stopping iterations when the objective function changed less than 1 × 10 − 4 over 20 iterations or after a maximum of 500 iterations, while maintaining stable non-significant differences (*p* > 0.05) over the last 20 iterations.

Initial model parameters, including priors, were set based on pilot experiments for each group of subjects. Visual and proprioceptive precisions were inversely proportional to the respective sensory noise variances (σ2v, σ2p). This simplified model does not include the vestibular sensory channel. The exclusion of the vestibular signal, which is involved in postural control, is recognized as a limitation of the existing modeling approach.

Optimization varied observation noise parameters for visual and proprioceptive channels, process noise, and control gain, while model structure, timestep, and state/observation dimensions were fixed.

Physiologically, __v represents visual precision weighting, __p proprioceptive precision weighting, process noise relates to motor variability, control gain corresponds to reflexive feedback, and sensory delay simulates afferent and efferent latencies.

## Results

**Fig. 2:**
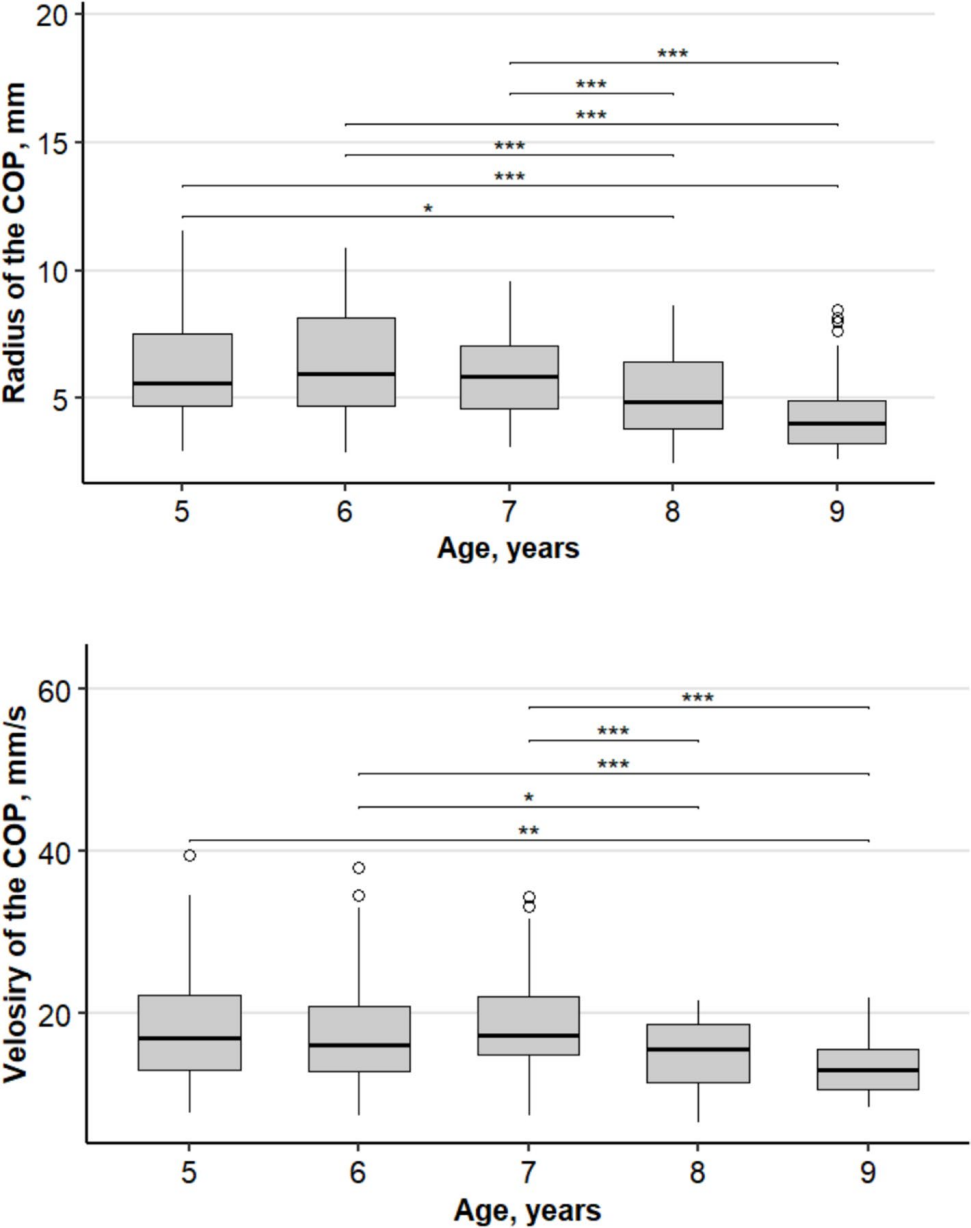
Dynamics of changes in the radius (left) and velocity of oscillation (right) of CoP in children aged 5–9 years old children engaged in three types of sports. Levels of significance of Student’s t-test: ***0.001, **0.01, *0.05. The box plot areas show the interquartile range (25–75%), the line is the median, and the whiskers are the minimum and maximum values, including outliers.. Age: 5 years—*n* = 98, 6 years—*n* = 148, 7 years—*n* = 154, 8 years—*n* = 169, 9 years—*n* = 123

Our experimental studies revealed clear age-related differences in the magnitude of postural oscillations during a natural stance (Fig. 2). The data are cross-sectional, and each age group is an independent sample (Student’s t-test for independent samples). In preschool-aged children (5–7 years), no significant differences were found (*p* > 0.05) in the studied parameters. After 7 years, the radius (*p* < 0.001) and velocity (*p* < 0.0001) of CoP oscillations in children begin to differ significantly from those in younger children. It should be noted that age-related parameters may be partially confounded by athletic experience.

**Fig. 3:**
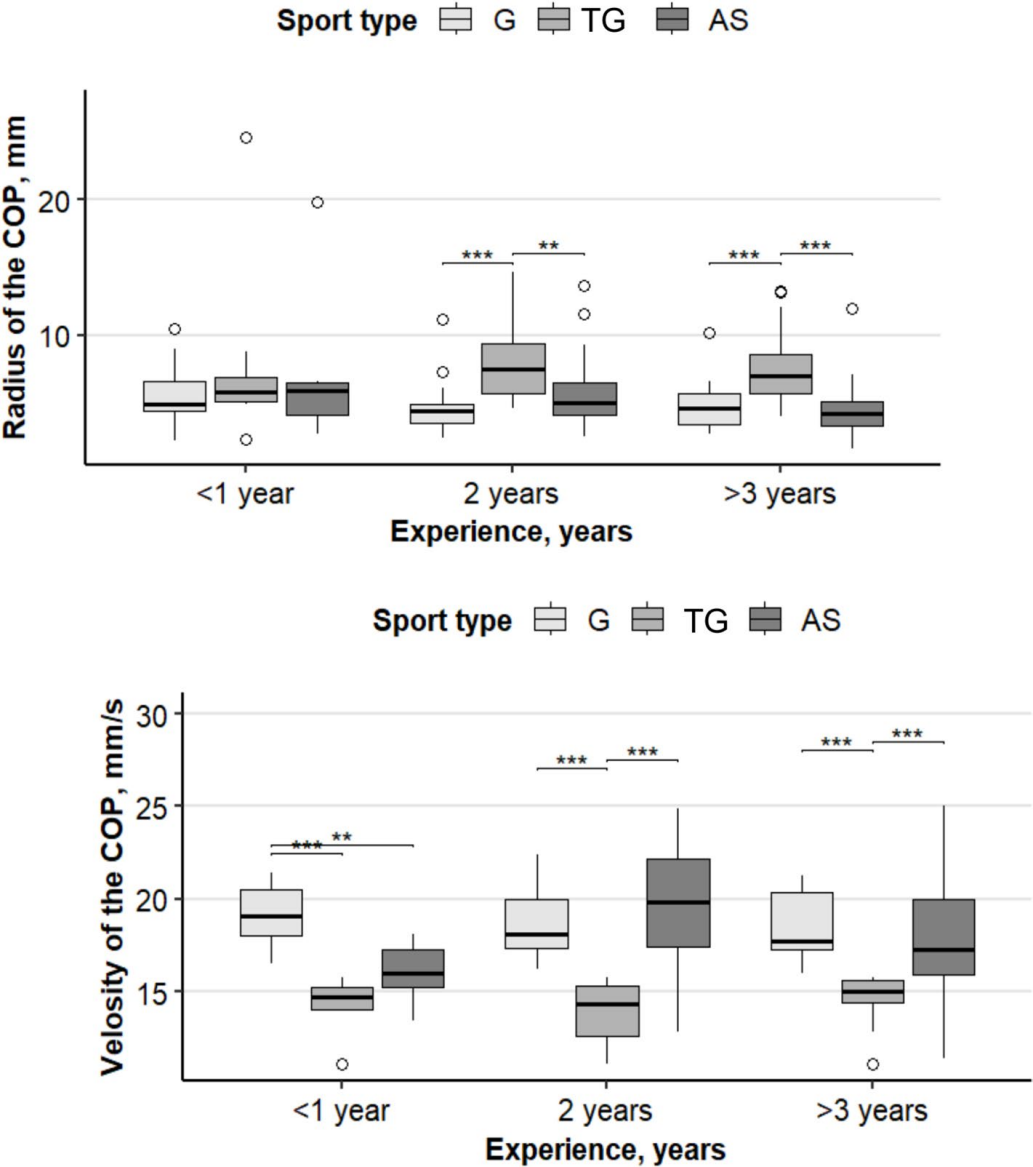
The influence of factors of the type of sport and the duration of sports on the indicators of radius (left) and speed (right) CoP at postural equilibrium. Levels of significance: ***0.001, **0.01, *0.05. The box plot areas show the interquartile range (25–75%), the line is the median, and the whiskers are the minimum and maximum values, including outliers. G—*n* = 209, TG—*n* = 245, AS—*n* = 238

The data presented in Fig. 3 show that the values of the studied indicators of postural stability have a certain pattern associated with the influence of practicing various sports. In the first year of classes, there was no significant difference (*p* > 0.05) between the children of the three specializations. Children who started playing their chosen sport at the age of 4–5 years after 3 years of practice were highly significantly different (*p* < 0.001) between groups of playing sports and gymnastics and between groups of playing sports and skiers. No significant difference was found (*p* > 0.05) by the third year of study between groups of gymnasts and skiers in terms of the CoP oscillation radius.

To investigate the specific effects of sport specialization while controlling for rapid neurodevelopmental changes, a two-way ANOVA was conducted on the 8-year-old cohort (M = 8.3, SD = 0.33 years). This age group was selected because (1) it represents children with sufficient training experience (≥ 3 years) to exhibit sport-specific adaptations, and (2) postural sway parameters show a relative plateau compared to the more volatile period of 5–7 years (Fig. 2), allowing for a clearer interpretation of sport-related effects.

Table 2 presents the results of a two-way analysis of variance (ANOVA) examining the effects of the duration of sport participation and the type of sport on stability performance in 8-year-old children (age M = 8.3, SD = 0.33 years).

**Table 2:** ANOVA results reflecting the influence of the length of study and type of sport activity on the 8-year-old cohort (M = 8.3, SD = 0.33 years) on the studied sustainability indicators (*n* = 169)

Parameter	Factor	Sum Sq	Mean Sq	F value	Pr(> F)
R, mm	Experience Sport type Experience: Sport type	23.6 3.7 51.3	23.57 3.66 51.31	2.574 0.399 5.605	0.1102 0.5281 0.0189 *
V, mm/s	Experience Sport type Experience: Sport type	2.6 0.2 47.1	2.59 0.17 47.15	0.275 0.018 5.005	0.6003 0.8936 0.0264 *

Levels of significance: * 0.05

All athletes were divided into three groups: those involved in the selected sport; as the first factor (experience), a group with up to 1 year of experience (*n* = 63), a group with 2 years of experience (*n* = 55), and a group with more than 3 years of experience (*n* = 51). The next factor (sport type) was the athletes’ affiliation with the selected sports specialization: TG (*n* = 60), G (*n* = 53), or AS (*n* = 56).

A significant interaction effect between experience and type of sport was observed for the radius of oscillation of the center of pressure (F = 5.605, *p* = 0.0189) and the speed of oscillation (F = 5.005, *p* = 0.0264). The main factors “experience” and “type of sport" alone did not show statistically significant differences.

After finding a significant interaction, simple main effects (with Bonferroni correction) were analyzed, and Tukey’s HSD post-hoc test was applied for subsequent pairwise comparisons. A significant difference was found for the velocity of oscillation of the center of pressure (V) between the gymnastics group and the team games group at the “2 years of experience” level (*p* = 0.0250). For the radius of oscillation of the center of pressure (R), a significant difference was found for the “more than 3 years of experience” level between the TG group and the AS group (*p* = 0.0220), as well as between the TG group and the G group (*p* = 0.0185). For the velocity of the center of pressure (V), a significant difference was found between the G group and the AS group at the “more than 3 years of experience” level (*p* = 0.0082), as well as between the TG group and G group at the “more than 3 years of experience” level (*p* = 0.0012). Other pairwise comparisons did not reach statistical significance.

The analysis of the modeling results presented in Table 3 allows us to compare the three groups according to the difference in internal generative models through the prism of the theory of active inference. The modeling was conducted using experimental data from 8-year-old children (M = 8.3, SD = 0.33 years) with at least 3 years of experience in the selected sport. Age and experience were determined based on the data in Table 2 and Figs. 2 and 3. The difference between the active inference model (AIF) indices from Table 3 and the specific measured values of R (radius) and V (velocity) from the experiment can be explained as follows. Free energy reflects the degree of discrepancy between the model-predicted postural control parameters and the actual measurements of R and V. Minimizing free energy means that the model adequately predicts the behavior of the COP, that is, it approaches the true values of the radius and velocity of sway. Complexity characterizes the complexity and variableness of the internal model of the agent required to explain the parameters R and V. Higher complexity may indicate a more variable control strategy based on the integration of different sensory data during balance maintenance. Accuracy measures the quality of the match between the predicted values (model R and V) and the experimental data. High accuracy means that the model successfully reproduces the dynamics of COP oscillations measured in the experiment. Posterior beliefs by position reflect the weighting of visual (position) and proprioceptive (velocity) information on the internal body model, i.e., how strongly the parameters R and V are involved in balance prediction and correction processes. Motor efficiency demonstrates the efficiency of generating motor corrections necessary to maintain specified values of R and V, reflecting the energy expenditure on compensating for deviations in position and velocity (CoP).

**Table 3:** Simulation results of agent control by a single-link inverted pendulum in accordance with the experimental data obtained (M ± m)

Indicator	Gymnastics *n* = 15	Team games *n* = 20	Alpine skiing *n* = 16
Free energy, c.u.*	4.758 ± 0.126	4.754 ± 0.127	4.974 ± 0.128
Complexity. c.u	3.169 ± 0.090	3.205 ± 0.096	3.423 ± 0.095
Accuracy. c.u	−1.589 ± 0.048	−1.549 ± 0.046	−1.551 ± 0.057
Posterior beliefs by position. c.u	12.269 ± 0.103	12.315 ± 0.085	12.298 ± 0.072
Posterior beliefs by speed. c.u	276.645 ± 5.233	273.050 ± 4.761	271.164 ± 6.223
Motor efficiency. c.u	1.185 ± 0.071	1.219 ± 0.067	1.180 ± 0.046

^*^ c.u.—Conventional units. Dimensionless values for relative comparison within the model

The team players, with a virtually identical free energy (Δ 0.08%, d = 0.065) compared to the gymnasts, demonstrated superior accuracy (Δ 2.58%, d = 2.74) and motor efficiency (Δ 2.87%, d = 1.089). A comparison with the alpine skiers revealed a significant decrease in free energy (Δ 4.62%, d = 2.724). indicating less efficient motor control in the alpine skiers. The alpine skiers’ high free energy is also determined by their relatively high accuracy (Δ 2.45%, d = 2,345) and complexity (Δ 6.80%, d = 2.281). Taken together, this leads to a significant difference in motor efficiency (Δ 3.31%, d = 1.678) between the alpine skiers and the team players.

## Discussion

The data in Fig. 2 clearly demonstrates age-related changes in the parameters of the radius and velocity of the center of pressure (CoP) oscillations in children involved in various sports, reflecting the process of postural stability development. The observed pattern in Fig. 2—small values of the radius of oscillation (R) in gymnasts and alpine skiers—indicates high precision of body position control in space, which is associated with the requirements of these sports for maintaining a stable and precisely controlled posture. At the same time, low values of the velocity parameter (V) in team sports athletes reflect a control strategy in which balance is achieved through smoother and slower adjustments, which is associated with the need for a quick reaction and adaptation to interactions with partners and opponents. Thus, gymnastics and alpine skiing require precise positional control, while team sports require dynamic and adaptive balance control. This demonstrates the influence of sports specialization and training experience on the development of the body schema and sensory integration, which is confirmed by the research of Panissa et al., 2017 [21]. The period of age-related changes of 7–8 years with nonlinear dynamics of parameters can be explained by the neurodevelopment of sensory and motor systems, which is confirmed by research in the field of motor development [22, 23].

Figure 3 shows significant changes in the range (R) and velocity (V) of the center of pressure (CoP) stability parameters depending on the type of sport and duration of training. No statistically significant changes were found between the parameters in the first year of training. which is consistent with the observations on a decrease in stability among novice athletes [21]. However, after three or more years of training, changes in the parameters are observed, which is supported by historical experience and sport specialization in the development of postural stability [24]. These results are also relevant to research on the adaptation of the sensorimotor system to the demands of different sports [25].

The data presented in Table 2 indicate that the interaction between sport type and duration of training experience significantly influences postural stability parameters in 8-year-old children. This suggests that the development of stability and postural control depends not only on the overall time spent playing sport but also on the specificity of a particular sport, highlighting the influence of sport specialization on the development of body schema and sensorimotor control [21, 25].

The lack of significant main effects for “experience” and “sport” separately highlights the need to consider their combined influence when interpreting the results. These data are consistent with the current understanding that adaptive changes in postural stability are driven by a complex interaction of training factors and the nature of motor activity [24]. The specific demands of each sport lead to the development of unique sensory integration and motor control strategies, which are reflected in the parameters of the radius and velocity of the center of pressure oscillation. This understanding has practical implications for sports pedagogy and coaching, emphasizing the importance of individualizing the training process based on both the athlete’s age and the specific sport discipline. This approach may facilitate more effective development of stability and balance skills in children [21].

Thus, our results support the need to consider the interaction between sport type and training experience when optimizing programs for developing effective sensorimotor balance control mechanisms and body schema in children.

However, experimental data do not explain the reasons underlying the improvement in postural stability control. Within the framework of the AIF, the quality of the body schema is defined fundamentally differently than it is in classical biomechanics. Here, the focus shifts from the description of stability to the accuracy of the brain’s predictions about the state of the body and its interaction with the world and the effectiveness of surprise minimization [6]. In the AIF paradigm. The quality of the body schema is the ability of the generative brain model to generate accurate predictions about the sensory consequences of the body state and its interaction with gravity and support. Our computer modeling of postural balance control in three groups of 8-year-old athletes with at least 3 years of training experience in different sports revealed that the groups differed in the balance of visual and proprioceptive accuracy, i.e., increased attention to the corresponding modality related to the task.

Representatives of the group of TG are characterized by optimal adaptation to solving motor tasks in comparison with athletes of other groups. A group of gymnasts demonstrates an imbalance between sensory integration modalities, possibly related to a predominance of visual information and a lesser reliance on proprioception. Because of this, spontaneous motor corrections occur due to distrust of the internal model. The inaccurate body layout of gymnasts is determined by errors in predicting the sensory effects of posture, which are compensated by motor hypercorrections.

It is assumed that, in the group of alpine skiers, the control system reacts late to changes in the speed of body movement. The visual analyzer predicts the movement of the marker on the screen with a delay, which leads to reliance on outdated proprioceptive data. This situation requires significant efforts (high-amplitude muscle activation), compared with other groups, to produce motor corrections. The inert body schema is characterized by insufficiently accurate predictions of changes in movement conditions, which leads to energy-intensive corrections.

The observed difference in the groups can be explained by the different needs for sensorimotor information associated with the performance of the main competitive exercise. Several studies have shown that the “dominant” modality depends on the context [26]. In our case, the context refers to external intervention in the form of a long-term training process. In other words, our results show that the updating of the “body model” predictions can be mediated by practicing a certain degree of sport specialization.

Our results support the theoretical model of sensory integration proposed by F. Horak (2006), which suggests that maintaining balance relies on the continuous integration of information from key sensory systems [3]. Each sensory system contributes to the formation of an internal representation of the body—the body schema—and helps the body navigate in space. Changes in the body schema are reflected in the parameters of postural sway, such as amplitude (the magnitude of the center of pressure deviation), velocity, and trajectory of sway. Based on Horak’s theory [3], it can be explained that improving or adapting the body schema through training experience reduces postural sway through more precise sensory integration and the effective use of information from various sensory systems.

This work has several limitations, since only three sports are considered, which is certainly insufficient and requires an increase, for example, the consideration of groups of combat sports and aesthetic sports. Additionally, a significant limitation is associated with the development of a simplified agent model associated with the control of a single-joint inverted pendulum. This model does not account for multi-joint movements and does not include information from the vestibular system. The cross-sectional design limits the ability to draw clear causal conclusions about the development of body schema and postural stability with age. A number of potential confounding factors, such as children’s daily activities, play, school physical education, and genetic factors, which may influence the development of postural control beyond the influence of sport, were not controlled for. However, it should be emphasized that these limitations do not detract from the value of the study and that its results are meaningful in real-world research settings.

Future research of interest includes conducting longitudinal studies with repeated measurements to examine the dynamics of body schema and postural control development as children grow and gain athletic experience; expanding the range of sports, including combat, aesthetic, and aquatic disciplines, to better understand the impact of sports specialization on sensorimotor adaptation and balance; and using more complex biomechanical models that take into account multi-joint movements and integrate the vestibular system to improve the theoretical framework and model postural control. Furthermore, neurophysiological research methods are being introduced to identify the mechanisms of sensory integration and predictive processing in balance control.

## Conclusion

In conclusion, our results indicate that the formation of the body schema in preschoolers and primary school children is closely related to multisensory integration and action. Representations of movement are based on a priori knowledge of the dynamics of movement and predicted sensory consequences of the action formed under the influence of various sports and reflecting the specific demands on the sensorimotor component inherent in the main competitive exercise.

We have shown that behavior in the task of maintaining postural balance changes in accordance with changes in the internal representation of the body configuration from the adjustment of the balance of visual and proprioceptive accuracy, i.e., increasing attention to the appropriate modality related to the solution of the motor task.

Our results generally support the formulation of predictive encoding of active inference, where visual and proprioceptive cues influence multimodal beliefs that drive action. The model experiment demonstrated that practicing a certain sport forms the appropriate proportions between the modalities determining the features of the internal body scheme of athletes of different specializations.

## Data Availability

The datasets used and/or analyzed during the current study are available from the corresponding author upon reasonable request.

## References

[1] Punakallio A. Balance abilities of different-aged workers in physically demanding jobs. J Occup Rehabil. 2003;13(1):33–43. 10.1023/a:1021845823521. 12611029 10.1023/a:1021845823521

[2] Mickle KJ, Munro BJ, Lord SR, Menz HB, Steele JR. Cross-sectional analysis of foot function, functional ability, and health-related quality of life in older people with disabling foot pain. Arthritis Care Res (Hoboken). 2011;63(11):1592–8. 10.1002/acr.20578. 22034121 10.1002/acr.20578

[3] Horak FB. Postural orientation and equilibrium: what do we need to know about neural control of balance to prevent falls? Age Ageing. 2006;35 Suppl 2:ii7-ii11. 10.1093/ageing/afl077. PMID: 16926210. 10.1093/ageing/afl077 16926210

[4] Kuo AD. An optimal control model for analyzing human postural balance. IEEE Trans Biomed Eng. 1995;42(1):87–101. 10.1109/10.362914. 7851935 10.1109/10.362914

[5] Fourneret P, Jeannerod M. Limited conscious monitoring of motor performance in normal subjects. Neuropsychologia. 1998;36(11):1133–40. 10.1016/s0028-3932(98)00006-2. 9842759 10.1016/s0028-3932(98)00006-2

[6] Friston K. The free-energy principle: a unified brain theory? Nat Rev Neurosci. 2010;11(2):127–38. 10.1038/nrn2787. 20068583 10.1038/nrn2787

[7] Kilner JM, Friston KJ, Frith CD. Predictive coding: an account of the mirror neuron system. Cogn Process. 2007;8(3):159–66. 10.1007/s10339-007-0170-2. 17429704 10.1007/s10339-007-0170-2 PMC2649419

[8] Pezzulo G, Rigoli F, Friston K. Hierarchical active inference: a theory of motivated control. Trends Cogn Sci. 2018;22(4):294–306. 29475638 10.1016/j.tics.2018.01.009 PMC5870049

[9] Friston K, Stephan KE. Free-energy and the brain. Synthese. 2007;159(3):417–58. 19325932 10.1007/s11229-007-9237-y PMC2660582

[10] Limanowski J, Friston K. Active inference under visuo-proprioceptive conflict: simulation and empirical results. Sci Rep. 2020;10(1):4010. 10.1038/s41598-020-61097-w. 32132646 10.1038/s41598-020-61097-w PMC7055248

[11] Friston K. Prediction. perception and agency. Int J Psychophysiol. 2012;83(2):248–52. 10.1016/j.ijpsycho.2011.11.014. 22178504 10.1016/j.ijpsycho.2011.11.014 PMC3314993

[12] Schulleri KH, Johannsen L, Michel Y, Lee D. Sex differences in the association of postural control with indirect measures of body representations. Sci Rep. 2022;12(1):4556. 10.1038/s41598-022-07738-8. (**PMID:35296686;PMCID:PMC8927351**). 35296686 10.1038/s41598-022-07738-8 PMC8927351

[13] Balan V, Mitrache G. Study in connection with the development of one’s body schema through the specific means of swimming. EpSBS. 2015;5(1):5–31. 10.15405/epsbs.2016.06.50

[14] Peterka RJ. Sensorimotor integration in human postural control. J Neurophysiol. 2002;88(3):1097–118. 10.1152/jn.2002.88.3.1097. 12205132 10.1152/jn.2002.88.3.1097

[15] Cicchella A. Static body balance in children and expert adults ballroom dancers: insights from spectral analysis of shifts. Biology. 2021;10(12):1291. 10.3390/biology10121291. 34943206 10.3390/biology10121291 PMC8698350

[16] Schwesig R. Kluttig A. Leuchte S. Becker S. Schmidt H. Esperer HD. Der Einfluss unterschiedlicher Sportarten auf die Haltungsregulation [The impact of different sports on posture regulation]. Sportverletz Sportschaden. 2009;23(3):148–54. German. 10.1055/s-0028-1109576. Epub 2009 Sep 11. PMID: 19750443.4 10.1055/s-0028-1109576 19750443

[17] Verbecque E, Vereeck L, Hallemans A. Postural sway in children: a literature review. Gait Posture. 2016;49:402–10. 10.1016/j.gaitpost.2016.08.003. 27505144 10.1016/j.gaitpost.2016.08.003

[18] Shestakov MP. Perception of sports movements. 2023. Taganrog: Izd-vo YFU. p. 334. 10.1234/yfu.book.2023.

[19] Kapteyn TS, Bles W, Njiokiktjien ChJ. Kodde L, Massen CH, Mol JM. Standartization in platform stabilometry being a part of posturography. Agressologie. 1983;24(7):321–6. PMID: 6638321. 6638321

[20] Priorelli M, Stoianov IP. Dynamic planning in hierarchical active inference. Neural Netw. 2025;185:107075. 10.1016/j.neunet.2024.107075. 39817980 10.1016/j.neunet.2024.107075

[21] Paniccia M, Wilson KE, Hunt A, Keightley M, Zabjek K, Taha T, et al. Postural stability in healthy child and youth athletes: the effect of age. sex. and concussion-related factors on performance. Sports Health. 2018;10(2):175–82. 10.1177/1941738117741651. 29131721 10.1177/1941738117741651 PMC5857732

[22] Hunt BAE, Wong SM, Vandewouw MM, Brookes MJ, Dunkley BT, Taylor MJ. Spatial and spectral trajectories in typical neurodevelopment from childhood to middle age. Netw Neurosci. 2019;3(2):497–520. 10.1162/netn_a_00077. (**PMID:30984904;PMCID:PMC6444935**). 30984904 10.1162/netn_a_00077 PMC6444935

[23] Lorås H, Sandseter EBH, Sando OJ, Storli L. Distinct clusters of movement entropy in children’s exploration of a virtual reality balance beam. Front Psychol. 2023;14:1227469. 10.3389/fpsyg.2023.1227469. 37915527 10.3389/fpsyg.2023.1227469 PMC10616470

[24] Jaworski J. Lech G. Witkowski K. Bujas P. Szczepanik K. Piepiora P. Influence of training and selection on postural stability and its relationship with sport level in judo practitioners aged 11–14 years. Front Psychol. 2023;13:1053426. 10.3389/fpsyg.2022.1053426. Published 2023 Jan 6. 10.3389/fpsyg.2022.1053426 PMC9853965 36687885

[25] Andreeva A, Melnikov A, Skvortsov D, Akhmerova K, Vavaev A, Golov A, et al. Postural stability in athletes: the role of sport direction. Gait Posture. 2021;89:120–5. 10.1016/j.gaitpost.2021.07.005. 34280882 10.1016/j.gaitpost.2021.07.005

[26] Rand MK, Heuer H. Visual and proprioceptive recalibrations after exposure to a visuomotor rotation. Eur J Neurosci. 2019;50(8):3296–310. 10.1111/ejn.14433. 31077463 10.1111/ejn.14433

